# YopE specific CD8^+^ T cells provide protection against systemic and mucosal *Yersinia pseudotuberculosis* infection

**DOI:** 10.1371/journal.pone.0172314

**Published:** 2017-02-16

**Authors:** Norberto González-Juarbe, Haiqian Shen, Molly A. Bergman, Carlos J. Orihuela, Peter H. Dube

**Affiliations:** 1 Department of Microbiology, The University of Alabama at Birmingham, Birmingham, Alabama, United states of America; 2 Department of Microbiology & Immunology, The University of Texas Health Science Center San Antonio, San Antonio, TX, United states of America; University of Helsinki, FINLAND

## Abstract

Prior studies indicated that CD8^+^ T cells responding to a surrogate single antigen expressed by *Y*. *pseudotuberculosis*, ovalbumin, were insufficient to protect against yersiniosis. Herein we tested the hypothesis that CD8^+^ T cells reactive to the natural *Yersinia* antigen YopE would be more effective at providing mucosal protection. We first confirmed that immunization with the attenuated *ksgA*^*-*^ strain of *Y*. *pseudotuberculosis* generated YopE-specific CD8^+^ T cells. These T cells were protective against challenge with virulent *Listeria monocytogenes* expressing secreted YopE. Mice immunized with an attenuated *L*. *monocytogenes* YopE^+^ strain generated large numbers of functional YopE-specific CD8^+^ T cells, and initially controlled a systemic challenge with virulent *Y*. *pseudotuberculosis*, yet eventually succumbed to yersiniosis. Mice vaccinated with a YopE peptide and cholera toxin vaccine generated robust T cell responses, providing protection to 60% of the mice challenged mucosally but failed to show complete protection against systemic infection with virulent *Y*. *pseudotuberculosis*. These studies demonstrate that vaccination with recombinant YopE vaccines can generate YopE-specific CD8^+^ T cells, that can provide significant mucosal protection but these cells are insufficient to provide sterilizing immunity against systemic *Y*. *pseudotuberculosis* infection. Our studies have implications for *Yersinia* vaccine development studies.

## Introduction

Three *Yersinia* species cause disease in humans: *Yersinia pestis*, *Yersinia enterocolitica* and *Yersinia pseudotuberculosis* [1]. Today, in the United States, *Y*. *pestis* infections occur mainly by inoculations from fleas carried by wild rodents [2], the last major plague outbreak in the US occurred in 1924 [3]. Ingestion of contaminated food or water is the source of enteric yersiniosis, caused by *Y*. *enterocolitica* or *Y*. *pseudotuberculosis* with swine being the most common source. This disease is characterized by fever, gastroenteritis and mesenteric lymphadenitis [4]. Albeit there are differences in routes of infection and disease severity, all *Yersinia* species are known to disseminate from lymphoid tissues to systemic organs. Many studies have demonstrated the ability of these pathogens to subvert host immune responses and the cellular death pathways that control bacterial replication leading to fulminant disease.

CD8^+^ T cells are known to be essential for the immune response against viruses; however, they also contribute as a line of defense against intracellular bacterial pathogens. Naïve CD8^+^ T cells detect infected cells by recognition of peptide antigens presented by the major histocompatibility complex (MHC) class I molecule on the surface of the cells [5]. Most of the peptide antigens presented by MHC class I molecules come from cytosolic proteins, for this reason pathogens or microorganisms that introduce antigens to the cytosol are subject to CD8^+^ T cell surveillance [6]. Along these lines, bacterial pathogens such as *Salmonella* spp., *Shigella* spp., *Vibrio cholerae*, and *Yersinia* use a virulence-associated molecular machine called a type III secretion system (T3SS) to directly inject or translocate bacterial toxins from the bacteria to the host-cell cytosol [7]. Using this mechanism, pathogenic *Yersinia* introduces multiple virulence factors known as *Yersinia* outer proteins (Yops) into the host cell. Yersinia spp. express six secreted Yops, these are: YopE, YopJ, YopH, YopM, YopO and YopT. These are known to disrupt cellular signaling pathways leading to changes in cytokine production and blockage of phagocytosis [8].

Since Yops are delivered directly to the cytosol by the T3SS, it is reasonable to expect CD8^+^ T cells might respond to Yop-derived antigens. In the last five years, multiple studies have shown that the T3SS effector protein YopE contains a dominant CD8^+^ T cell epitope specifically recognized by CD8^+^ T cells isolated from *Y*. *pestis* or *Y*. *pseudotuberculosis* infected or immunized mice [9–12]. YopE, a Rho GTPase-activation protein (GAP), is characterized as a contact-dependent cytotoxin, responsible for inhibition of phagocytic processes by disruption of the actin cytoskeleton [13]. YopE amino acids 69 to 77 (YopE_69-77_) have been shown to be a dominant epitope recognized by CD8^+^ T cells in C57BL/6 mice and immunization with peptides containing the epitope confers significant protection from lethal pulmonary challenge with *Y*. *pestis* [10].

A recent publication by Zhang et al. showed that effector CD8^+^ T cells were generated in response to *Y*. *pseudotuberculosis* YopE_69-77_. Moreover, that the YopE_69-77_ specific CD8^+^ T cells produced IFN-γ and TNF-α 14 days after infection with a YopE GAP mutant and that these cells were positive for KLRG1, a marker associated with the memory phenotype [14]. The latter report in combination with our own [9, 11], suggests that vaccination with an attenuated version of *Y*. *pseudotuberculosis* induces a protective antigen-specific CD8^+^ T-cell response against the wild-type infection. Herein, we test the hypothesis that CD8^+^ T cells specific to the natural *Yersinia* antigen YopE would be effective at protecting the host against mucosal *Y*. *pseudotuberculosis* infections. We show that mice immunization with *Listeria monocytogenes* expressing YopE or cholera toxin in addition to a YopE_69-77_ peptide resulted in the development of varying degrees of YopE-specific CD8^+^ T cell protection against *Y*. *pseudotuberculosis* systemic or mucosal infection. These data demonstrate that cholera toxin in combination with YopE_69-77_ is sufficient to generate partial protection (≥60%) to *Y*. *pseudotuberculosis* after oral challenge and significantly extend the survival of systemically infected mice.

## Materials and methods

### Mouse strains and procedures

All animal use procedures were performed in strict accordance with the NIH Guide for the Care and Use of Laboratory Animals and were approved by the Institutional Animal Care at the University of Texas Health Sciences Center San Antonio (Protocol #12030X). C57BL/6 mice were obtained from Charles River (Wilmington, MA). Female 8–10 week-old mice were used for all experiments and were allowed to rest for 7 days after arrival prior to use. Mice were housed in specific-pathogen-free conditions in isolator cages with soft bedding. Mice were given free access to food, standard mouse chow, and water throughout the course of the experiment. Animals were cared for by department of laboratory animal staff. During experimental procedures animals were monitored by laboratory staff twice a day and there were no unexpected deaths during this study. Animals in survival studies were considered morbibund if they lost 20% of their body weight and were subsequently euthanized by isoflurane overdose. Animal suffering was minimized by providing free access to food and water, animals had soft bedding for the duration of the experiments and all procedures were done under general anesthesia, 3% isoflurane and oxygen.

### Bacterial strains (Table 1)

The *Yersinia pseudotuberculosis* YPIII and isogenic *ksgA*^*-*^ strain have been described and used by us previously [15, 16]. The *ksgA*^*-*^ strain contains a kanamycin-resistance-encoding transposon inserted into the *ksgA* gene. The attenuated *L*. *monocytogenes* Δ*actA* Δ*plcB* strain in the 10403S background [17], was used as a vaccine vector in these studies. *L*. *monocytogenes* expressing a secretable YopE-OVA fusion protein was constructed as previously described by Lauer et al. by inserting the YopE fragment in-between a sequence encoding the *actA* promoter and the first 100 amino acids of the ActA protein and a sequence encoding OVA_254-265_ in the *L*. *monocytogenes* integration vector pPL2 [18]. The construct was stably integrated at the tRNA^Arg^ locus of the bacterial chromosome of the attenuated *Listeria monocytogenes* Δ*actA* Δ*plcB* strain, as described previously [19].

**Table 1:** 

DH5 α		F^-^ Δ(*lacZYA-argF*) *U169 recA1 endA1 hsdR17 supE44thi-1 gyrA96 relA1*
DH5 α λpir		FΔ(*lacZYA-argF*) *U169 recA1 endA1 hsdR17 supE44thi-1 gyrA96 relA1 λ*::*pir*
SM10λpir		*thi recA thr leu tonA lacY supE RP4-2-Tc*::*Muλ*::*pir*
Stb12/pVEV		F^-^ *mcr*A Δ*(mcrBC-hsdRMS-mrr) recA1 endA1 lon gyrA96 thi supE44 relA1* Δ(*lacproAB*)/pVEV
*Yersinia pseudotuberculosis* strains (all in YPIII/pIB1 background)		
JM500	*ksgA*^*-*^	
MB323	*ksgA*^*-*^::pCVD442-*yopE*_*1-138*_*-ova*_*247-355*_	
MB327	Δ*yopK*	
*Listeria monocytogenes* strains		
10403s	wildtype	
JJL-OVA	10403s::*actA*_*ss*_-*ova*_*1-386*_	
LH1169	10403s Δ*actA* Δ*plcB*	
DH-1558	10403s Δ*actA* Δ*plcB*::pVEV (*actA*_*1-100*_*-ova*_*254-266*_)	
MB297	10403s::pHQS1 (*actA*_*1-100*_*-yopE*_*1-219*_*ova*_*254-266*_)	
MB301	10403s::pHQS2 (*actA*_*1-100*_*-yopE*_*64-82*_*ova*_*254-266*_)	
MB302	10403s Δ*actA* Δ*plcB*::pHQS1 (*actA*_*1-100*_*-yopE*_*1-219*_*ova*_*254-266*_)	
MB304	10403sΔ*actA* Δ*plcB*::pHQS2 (*actA*_*1-100*_*-yop*_*64-82*_*Eova*_*254-266*_)	

### Flow cytometry

For spleen single cell suspensions the tissue was gently dissociated with a syringe plunger through Falcon® 70 μm Cell Strainer (BD Biosciences, San Jose CA). After lysing the erythrocytes with PharmLyse (BD Biosciences, San Jose CA), approximately 1 × 10^6^ cells were blocked with FcBlock (clone 2.4G2) for 15 min on ice. Then cells were stained with antibodies (1:1000 dilution) to murine CD4 (L3T4, clone RM4-4, FITC conjugated), CD8 (Ly-2, clone 53–6.7, PE or FITC conjugated), CD3e (IgG1, clone 145-2C11, PerCP-Cy5.5 conjugated), and CD45 (IgG2b, clone 30-F11, APC conjugated) for 30 minutes on ice covered from direct light. After staining cells were fixed with 2% paraformaldehyde (Electron Microscopy Services, Hatfield PA), and analyzed using a FACSCalibur (BD Biosciences Immunocytometry Systems, San Diego CA). The SIINFEKL Pentamer and a control unloaded pentamer labeled with APC were purchased from Proimmune (Proimmune, Sarasota, FL). A MHC class I H2-Kb tetramer loaded with YopE_69–77_ (K^b^YopE_69–77_) was obtained from the NIH tetramer core. Pentamers and tetramers were used at a dilution of 1:500 and staining was done as described for antibody staining for flow cytometry.

### Cellular stimulation and intracellular cytokine staining

T-cell stimulation and intracellular cytokine staining was done essentially as we have previously described [20]. Splenocytes from animals in the indicated treatment groups were harvested and stimulated in vitro with 1μg of OVA_257-264_ peptide, YopE_69-77_ peptide, or the saline vehicle as a control for 24 hours. As a positive control cells were non-specifically stimulated with plate-bound anti-CD3e (1μg/well) and costimulation was provided by treatment with 500ng/well anti-CD28. Cells were then stained with fluorochrome-conjugated antibodies against CD3e, CD8, and IFN- γ. Data was collected and analyzed on a FACSCalibur. Briefly, lymphocytes were identified by light scattering and CD3+ cells were gated to facilitate the analysis of IFN- γ expressing CD8+ cells.

### Antibody-mediated CD8+ cell depletion *in vivo*

T-cells were deleted as we have previously described [20, 21]. Briefly, mice were injected IP with 200μg CD8+ cell depleting antibodies (IgG2b, clone 2.43) or an isotype control anti-KLH antibody (IgG2b, clone LFT-2). Antibodies were injected on day -2 prior to challenge and then every four days. CD8+ T-cell depletion was confirmed by flow cytometry after gating on CD45+ cells (IgG2b, clone 30-F11, APC conjugated) and then analyzing CD8+ (Ly-2, clone 53–6.7, PE conjugated) and CD4+ (L3T4, clone RM4-4, FITC conjugated) cells on a LSRII (BD Biosciences Immunocytometry Systems, San Diego CA). Data was analyzed on the FloJo suite V.10.

### *In vivo* inoculations, immunizations, survival and weight change assays

*Y*. *pseudotuberculosis* strains were grown as follows, after 2 days growth on LB agar plates, single colonies were inoculated into 2 mL LB broth and incubated o/n at 26°C with rotation. Overnight cultures were then diluted to the desired concentration in PBS and used for mouse challenges as described below. *L*. *monocytogenes* strains were grown overnight in brain-heart infusion broth at 30°C without rotation, and diluted in PBS to the desired concentration and delivered intravenously. Mice were then visually inspected for signs of illness including lethargy and piloerection. For immunization studies, mice were allowed to rest for the indicated amount of time prior to challenge with the indicated bacteria. For most experiments, mice were challenged sixty days after immunization to allow time for the primary immune response to resolve and memory to be established. Mouse morbidity, weight change and mortality were recorded over a four to six-week time course. **Mucosal immunization**: on days 0, 7, and 21, mice were anesthetized with isoflurane and immunized intranasally with 10 μg YopE_69–77_ peptide mixed with 1μg of Cholera toxin (CT; List Biological Laboratories Inc., Campbell, CA) in 15 μL of PBS. Control mice received CT alone. On day 44, mice were challenged as indicated.

### Detection of Ag presentation by LacZ T cell hybridoma assay

2.0 X 10^5^ macrophages were incubated with *L*. *monocytogenes* expressing the ActA-YopE-OVA fusions, control bacteria expressing secreted OVA, the parental OVA-negative strain, or synthetic SIINFEKL peptide (i.e. OVA_257-264_) at the indicated concentrations for 12 hours. B3Z T cell hybridomas were co-incubated with the infected macrophages for 18 hours. B3Z responses were analyzed using a LacZ T cell hybridoma assay. After incubation, PBS was used to wash the plates and LacZ buffer and substrate (Roche) was added and cells were further incubated for up to 4 hours. LacZ enzymatic activity was measured by changes in the absorbance of a colorimetric substrate at 570 nm using a BioTek Synergy H4 plate reader.

### Data and statistical analysis

Flow cytometry data was analyzed with FlowJo (Tree Star, Ashland OR). Graphing and statistical analysis was performed with Prism (GraphPad Software, La Jolla CA). Kaplan Meier method was used for survival curves and significance calculated using the log-rank test. The nonparametric Mann-Whitney *U* test was used to determine statistical differences between groups of animals.

## Results

### Systemic immunization with attenuated *Y*. *pseudotuberculosis* generates YopE_69-77_-specific CD8^+^ T cells

**Fig 1:**
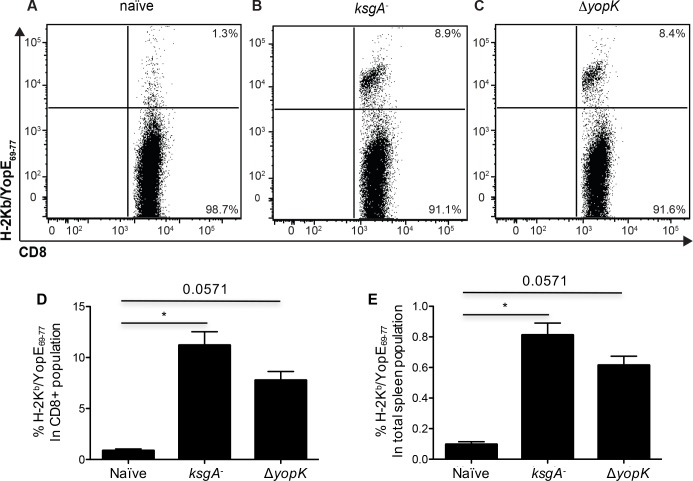
Generation of YopE_69-77_-specific CD8^+^ T cells in C57BL/6 mice exposed to attenuated *Y*. *pseudotuberculosis* strains. C57BL/6 mice were intravenously inoculated with *ksgA*^*-*^ (10^2^ CFU) or Δ*yopK* (10^3^ CFU) bacteria, then sacrificed on day 8 and splenic cells analyzed by flow cytometry. (**A-C**) YopE_69-77_-specific CD8^+^ T cells were detected by Kb/YopE_69-77_ tetramer staining, as shown in representative FACS plots after gating for CD3^+^, CD4^-^ and CD8^+^ cells. The percent of Kb/YopE_69-77_ tetramer^+^ cells in the CD8^+^ T cell population (**E**) is shown, with bars indicating mean values + S.E.M. The data are representative of two independent experiments (n = 4–5 mice). For multiple group comparisons Dunn’s multiple-comparison post-test was used: *, P ≤ 0.05, **, P ≤ 0.01, ***, P ≤ 0.001, ****, P ≤ 0.0001.

A protective immune responses to virulent *Y*. *pseudotuberculosis* can be generated by immunization with an attenuated *Y*. *pseudotuberculosis* strain lacking the 16s rRNA methyltransferase KsgA, and that protection required CD8^+^ T cells [9]. To determine if immunization with *ksgA*^*-*^ *Y*. *pseudotuberculosis* stimulated YopE_69-77_-specific CD8^+^ T cells, we evaluated the frequency of K^b^ YopE_69–77_ tetramer-positive CD8^+^ T cells at day 8 post-intravenous inoculation with 200 CFU of *ksgA*^*-*^ bacteria. Ten percent of spleen CD8^+^ T cells from *ksgA*^*-*^ immunized animals showed positive staining for the K^b^ YopE_69–77_ tetramer, in contrast to the <2% of spleen CD8^+^ T cells from naïve mice (Fig 1A, 1B, 1D and 1E). To test if the amount of YopE translocated into host cells limited CD8^+^ T-cell responses, we also tested a *yopK* mutant. A *Y*. *pseudotuberculosis* strain lacking the T3SS protein YopK is attenuated for virulence and hypertranslocates Yops into host cells [22–24]. We found that the frequency of K^b^ YopE_69–77_ tetramer^+^ CD8^+^ T cells in spleens was unaffected by the absence of YopK (Fig 1C and 1E). These results affirm that mice exposed to attenuated *Y*. *pseudotuberculosis* via the systemic route generate splenic T cells against the YopE_69-77_ epitope.

### YopE_69-77_-specific CD8^+^ T cells generated by attenuated *Y*. *pseudotuberculosis* infection are sufficient to protect against virulent rYopE-expressing *Listeria monocytogenes*

**Fig 2:**
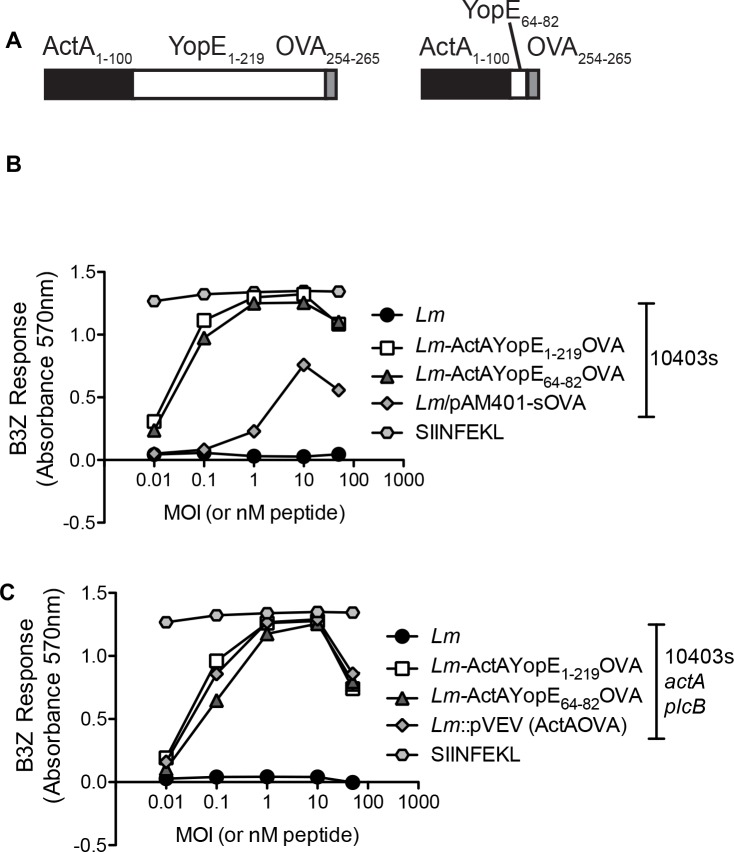
Construction of recombinant *L*. *monocytogenes* expressing a secretable YopE-OVA fusion protein and impact of YopE sequence on acquisition/processing/presentation of OVA_257-264_ peptide to OVA-specific CD8^+^ T cells. (**A**) Cartoon of engineered fusion proteins. Sequence encoding either full-length YopE (amino acids 1–219) or truncated YopE (amino acids 64–82) was inserted in-between sequence encoding the *actA* promoter and the first 100 amino acids of the ActA protein and sequence encoding OVA_254-265_ in the *L*. *monocytogenes* integration vector pPL2 (Lauer et. al. 2002, see methods). Resulting plasmids then were integrated into the *L*. *monocytogenes* genome. (**B, C**) Macrophages were incubated with *L*. *monocytogenes* expressing the ActA-YopE-OVA fusions, control bacteria expressing secreted OVA, the parental OVA-negative strain, or synthetic SIINFEKL peptide (i.e. OVA_257-264_) at the indicated concentrations. After 12 hours, macrophages were incubated with B3Z T cells, a T cell hybridoma activated by the recognition of H-2K^b^ in association with OVA_257–264_ peptide, and T cell activation measured using LacZ T cell hybridoma assay. Strain backgrounds were either the mouse-virulent 10403S (**B**) or mouse-attenuated 10403S Δ*actA* Δ*plcB* (**C**). Data is representative of two separate experiments. For multiple group comparisons Dunn’s multiple-comparison post-test was used: *, P ≤ 0.05, **, P ≤ 0.01.

We exploited the fact that CD8^+^ T cells are critical for protecting hosts against the cytosolic pathogen *L*. *monocytogenes*, and engineered this pathogen to express and secrete YopE. We inserted either full-length YopE or YopE_64-82_ downstream of the first 100 amino acids of the *Listeria* protein ActA, to allow for secretion of the fusion protein, and upstream of a C-terminal OVA_254-265_ tag, to allow for detection of antigen delivery/processing/presentation (Fig 2A). Macrophages were infected with the different *L*. *monocytogenes* strains and then incubated with B3Z cells, a T cell hybridoma that is activated by the recognition of H-2K^b^ in association with OVA_257–264_ peptide. Both ActA-YopE-OVA and ActA-YopE_64-82_-OVA were capable of being processed and presented by infected macrophages to stimulate B3Z cells equivalently or better than *L*. *monocytogenes* expressing OVA from a chromosome-integrated (Fig 2B) or plasmid-borne allele (Fig 2C). These results demonstrate that insertion of YopE into the ActA-OVA fusion protein does not interfere with antigen processing and presentation to responding T cell hybridomas, and suggests that the YopE portion of the fusion will be correctly processed and presented.

**Fig 3:**
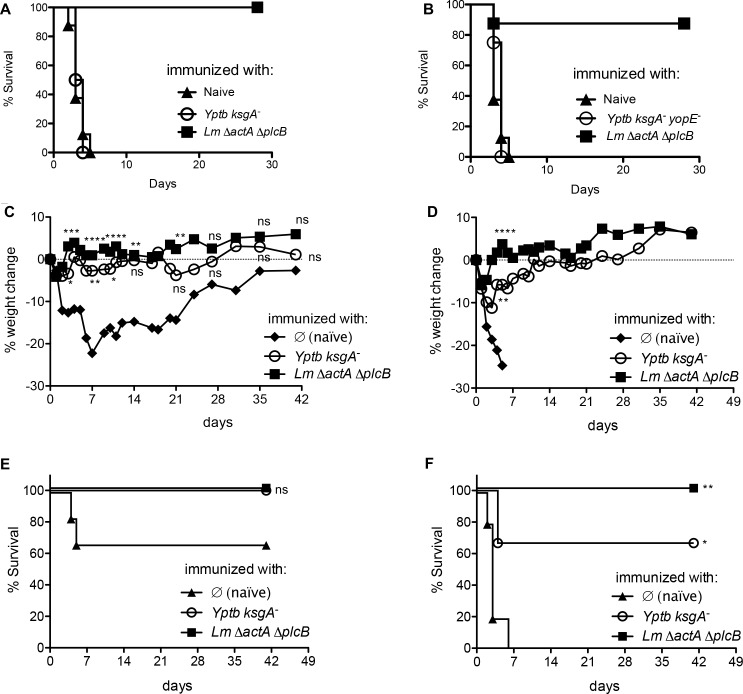
*ksgA*^*-*^ immunization protects against recombinant *L*. *monocytogenes* expressing YopE. 60 days after intravenous immunization with 200 CFU *ksgA*^*-*^ *Y*. *pseudotuberculosis* (*Yptb*), 200 CFU *ksgA*^*-*^ *yopE*^*-*^ *Yptb*, or 5.0 x 10^7^ CFU Δ*actA* Δ*plcB L*. *monocytogenes* (*Lm*) or nothing (naïve), C57BL/6 mice were intravenously challenged with either 5.0 x 10^5^ CFU Lm 1043s **(A)** 5.0 x 10^5^ CFU (**C, E**) or 2.0 x 10^6^ CFU (**B, D, F**) of virulent *L*. *monocytogenes* expressing ActAYopE_1-219_OVA and followed for mortality (% survival **(A, B, E, F**) or morbidity (% weight change, **C, D**) over a four to six week time course. The data are representative of two independent experiments (n = 4–6 mice/experiment **C, D, E**, and **F**) or a single experiment (n = 8 mice/group **A, B**). For multiple group comparisons Dunn’s multiple-comparison post-test was used for weight change differences at selected time points: *, P ≤ 0.05, **, P ≤ 0.01, ***, P ≤ 0.001, ****, P ≤ 0.0001. Log rank Mantel-Cox test was used for survival curves.

Initially we immunized mice IV with 200 CFU of the *ksgA* mutant or the *ksgA yopE* double mutant and once memory was established sixty days later, we tested susceptibility to challenge with 5.0 x 10^5^ CFU of the wild type parental *L*. *monocytogenes* strain, 1043s (Fig 3A), or *L*. *monocytogenes* ActAYopE_1-219_ (Fig 3B). The *ksgA*^*-*^ strain provides no protection against virulent *L*. *monocytogenes* challenge. Subsequently, to determine if infection with the *ksgA*^*-*^ mutant conferred YopE-mediated protection against infection, we immunized C57BL/6 mice with 200 CFU of the *Y*. *pseudotuberculosis ksgA-* strain or the attenuated *L*. *monocytogenes* Δ*actA* Δ*plcB* mutant and 60 days after, mice were challenged with a low dose of 5 x 10^5^ CFU (Fig 3C and 3E) or a high dose of 2.0 x 10^6^ CFU (Fig 3D and 3F) of virulent *L*. *monocytogenes* expressing the ActAYopE_1-219_ fusion protein_._ No significant weight loss or decrease survival was observed between the *Y*. *pseudotuberculosis ksgA- and L*. *monocytogenes* Δ*actA* Δ*plcB* immunized mice after low dose *L*. *monocytogenes* ActAYopE_1-219_ challenge, however, ~20% weight loss and ~60% survival was observed 1 week after infection in the control mice (Fig 3C and 3D). When mice were challenged with the high dose of *L*. *monocytogenes* ActAYopE_1-219_ a maximum of 10% weight loss was observed in mice immunized with *Y*. *pseudotuberculosis ksgA-*, while ~75% of the cohort survived the infection in comparison to 0 percent survival in control mice (Fig 3B and 3D). These results show that the host response generated against *Y*. *pseudotuberculosis ksgA-* was sufficient to protect against virulent *L*. *monocytogenes* in a YopE-specific manner.

### Immunization with attenuated *L*. *monocytogenes* expressing YopE results in the generation of YopE-specific CD8^+^ T cells

**Fig 4:**
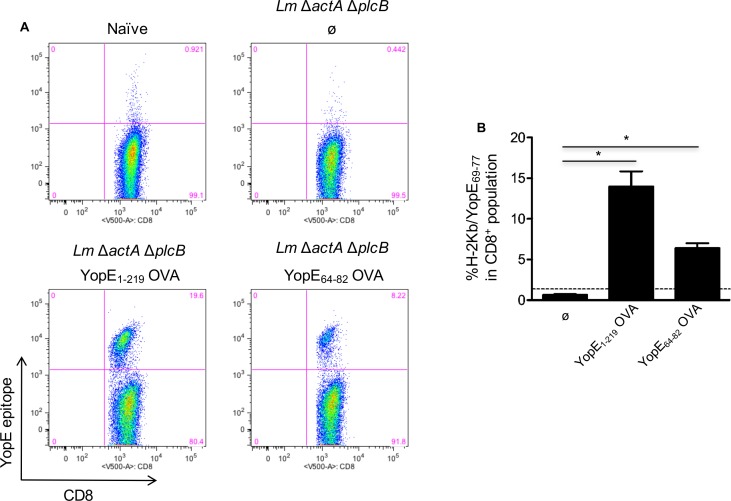
Mice immunized with attenuated YopE^+^ *L*. *monocytogenes* strains generate YopE_69-77_-specific CD8^+^ T cells. C57BL/6 mice were intravenously inoculated with 5.0 x 10^7^ CFU of the attenuated *Lm* Δ*actA* Δ*plcB* strains, either expressing no OVA (Ø) or the indicated ActAYopEOVA fusions, or left naïve, and spleen cells analyzed day 8 post-inoculation by flow cytometry. YopE_69-77_-specific CD8^+^ T cells were detected by K^b^ YopE_69-77_ tetramer staining, and the % of K^b^ YopE_69-77_ tetramer^+^ cells in the CD8^+^ T cell population (**A**). Quantitative representation of CD8^+^ T cell population percentages (**B**). Bars indicating mean values + S.E.M., and the data are representative of two independent experiments (n = 4–5 mice/experiment). Doted line represents naïve mice percentage. For multiple group comparisons Dunn’s multiple-comparison post-test was used: *, P ≤ 0.05.

**Fig 5:**
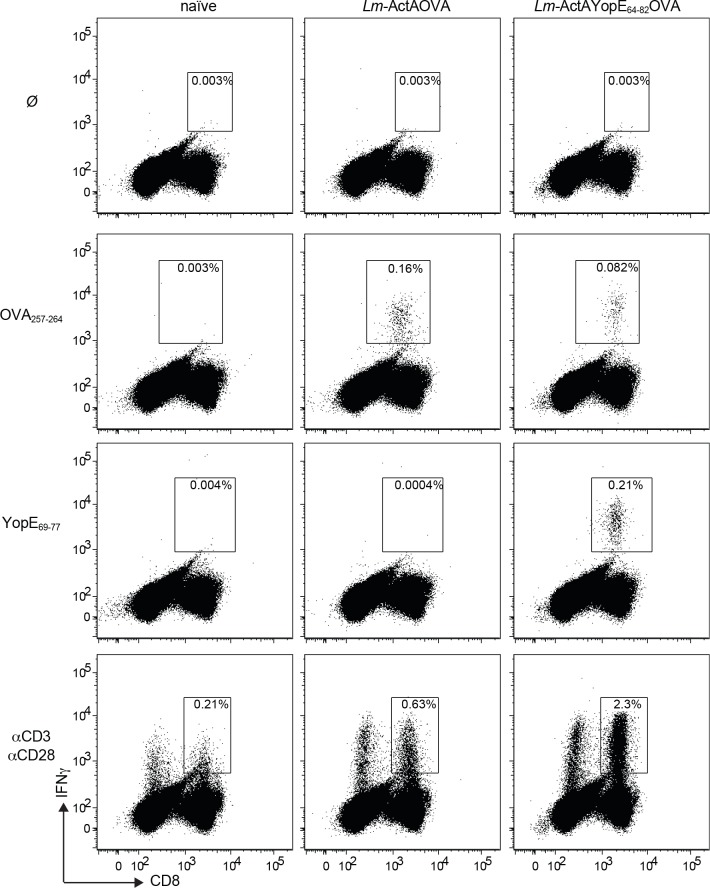
Murine immunization with attenuated *L*. *monocytogenes* expressing the YopE epitope generates functional YopE-specific CD8^+^ T cells. C57BL/6 mice were immunized with 5.0 x 10^7^ CFU of Δ*actA* Δ*plcB L*. *monocytogenes* strains expressing the indicated fusion proteins. 8 days later, spleen cells were stimulated with OVA_257-264_ peptide, YopE_69-77_ peptide, or antibodies against CD3 and CD28 and stained for intracellular IFN-γ. Representative histograms are shown from one of two separate experiments. The data are representative of two independent experiments (n = 3–5 mice/experiment).

To further analyze the ability of YopE^+^ *L*. *monocytogenes* to generate YopE-specific CD8^+^ T cells, we immunized mice with *L*. *monocytogenes* Δ*actA*Δ*plcB*, *L*. *monocytogenes ActAYopE*_*1-219*_ *OVA*, *L*. *monocytogenes ActAYopE*_*64-82*_ OVA or left naïve (Fig 4). Mice immunized with *L*. *monocytogenes ActAYopE*_*1-219*_ OVA had from 14–19% YopE positive CD8^+^ T cells, whereas mice immunized with *L*. *monocytogenes ActAYopE*_*64-82*_ OVA showed only 7–8% YopE positive CD8^+^ T cells, however both groups had significantly more YopE positive CD8^+^ T cells that the mice immunized with the *L*. *monocytogenes* Δ*actA*Δ*plcB* parental strain (Fig 4B). To assess the functionality of the *L*. *monocytogenes* generated YopE-specific CD8^+^ T cells, we immunized mice with *L*. *monocytogenes* Δ*actA*Δ*plcB*, *L*. *monocytogenes ActAYopE*_*64-82*_ OVA or left naïve. Eight days later, spleen cells were isolated and then stimulated with OVA_257-264_ peptide, YopE_69-77_ peptide, or antibodies against CD3 and CD28 and stained for interferon gamma (IFN-γ) (Fig 5). We observed that mice immunized with *L*. *monocytogenes ActAYopE*_*64-82*_ OVA only responded to the YopE_69-77_ peptide and the antibodies against CD3 and CD28 suggesting the generation of functional YopE specific CD8^+^ T cells (Fig 5).

### Immunization with attenuated *L*. *monocytogenes* expressing an YopE epitope mediates CD8+ T-cell protection against lethal *Y*. *pseudotuberculosis* challenge

**Fig 6:**
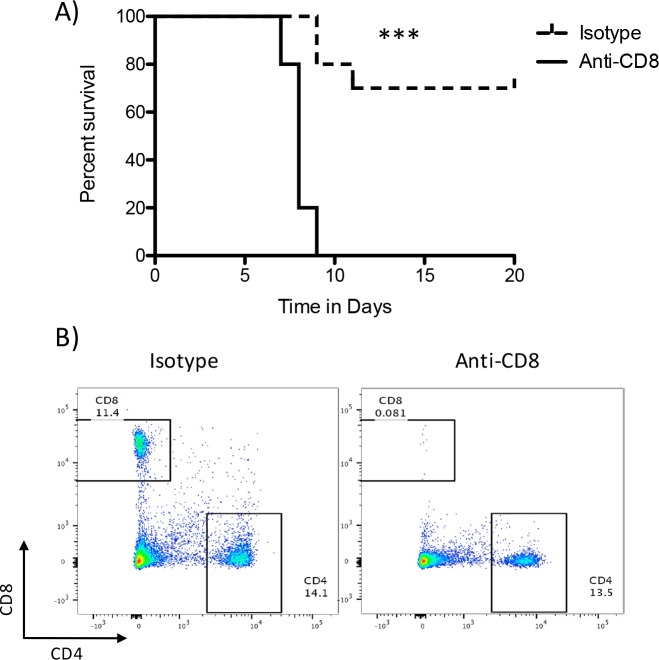
Immunization-mediated protection requires CD8+ T-cells. Mice (20) were immunized IV with 5.5 X 10^7^ CFU *L*. *monocytogenes ActAYopE*_*1-219*_ *OVA* and allowed to rest for 60 days. Mice were then treated IP with 200μg of anti-CD8 (2.43) or an isotype control (rat IgG2b LTF-2) two days prior to challenge and then every four days for the course of the experiment. Mice (9 from each treatment group) were challenged by oral gavage with 9 X10^8^ CFU of *Y*. *pseudotuberculosis* YPIII/pIB1 and survival was monitored for 20 days. **A)** Kaplan-Meier survival curve. Animals depleted of CD8 T-cells are represented by the solid line and those treated with the isotype control antibody by the dashed line. (p<0.0001, Mantel-Cox log-rank analysis). **B)** One mouse from each treatment group was analyzed by flow cytometry prior to challenge to ensure CD8+ cell depletion. Splenocytes were stained for CD45, CD4, and CD8. CD45+ cells were gated on and analyzed for the expression of CD4 and CD8. Essentially all CD45+ CD8+ cells were depleted with antibody treatment. These experiments were done once.

To test the requirement for CD8^+^ T cells for protection against *Yersinia* following *L*. *monocytogenes* immunization, we immunized mice with 5.5 X 10^7^ CFU *L*. *monocytogenes ActAYopE*_*1-219*_ *OVA* IV, and then allowed the animals to rest for 60 days. Mice were then treated IP with 200μg of a CD8 depleting antibody or an isotype control antibody two days prior to oral challenge with 9 X 10^8^ CFU of *Y*. *pseudotuberculosis* YPIII and then every four days throughout the course of the experiment. As shown in Fig 6A, protection following *L*. *monocytogenes* immunization required CD8+ T-cells. All animals treated with CD8 neutralizing antibodies succumbed to challenge with *Y*. *pseudotuberculosis* within 10 days whereas 70% of animals treated with an isotype control antibody survived the 20 day experiment (p = 0.0001). To confirm effective CD8 T-cell depletion, spleens were harvested from an animal treated with the isotype control antibody and the depleting antibodies prior to challenge with *Yersinia*. Single cell suspensions were stained with fluorescent antibodies for CD45, CD4, and CD8 an then analyzed by flow cytometry. CD45+ cells were gated on and then CD4 and CD8 expression was analyzed. Treatment with the anti-CD8 antibody eliminated CD8+ cells from the spleen (Fig 6B).

### Prior exposure to YopE confers partial protection against lethal *Y*. *pseudotuberculosis* oral and systemic infection

**Fig 7:**
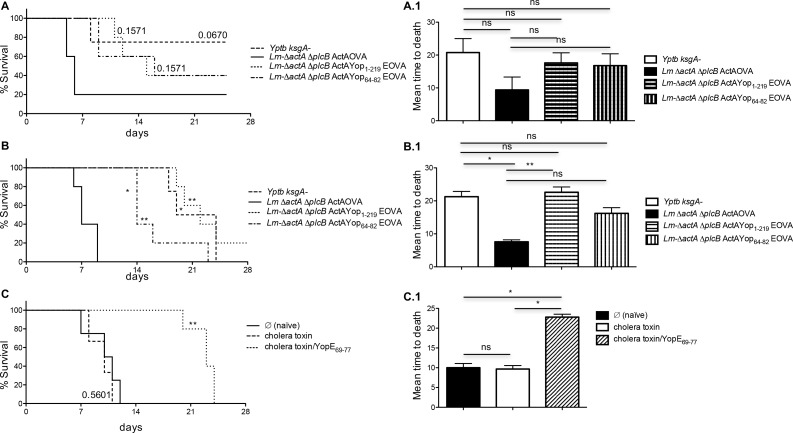
YopE-specific CD8^+^ T cells provide partial protection against virulent *Y*. *pseudotuberculosis* challenge via systemic or mucosal routes. 60 days after immunization with either attenuated YopE-expressing bacteria (**A, A.1, B, C.1**) or cholera toxin plus YopE_69-77_ peptide **(C, C.1**), mice were challenged with *Y*. *pseudotuberculosis* strain YPIII/pIB1 (fully mouse virulent), either 10^9^ CFU delivered orally (**A**) or 200 CFU delivered intravenously (**B, C**). Percent survival followed over a six week time course (A, B, C), mean time to death (A.1, B.1, C.1). Data are representative of two independent experiments (n = 3–6 mice/experiment). Log rank Mantel-Cox test was used for survival curves. For multiple group comparisons Dunn’s multiple-comparison post-test was used: *, P ≤ 0.05, **, P ≤ 0.01.

To test if induction of functional YopE-specific CD8^+^ T cells protected against virulent *Y*. *pseudotuberculosis*, we immunized mice with either attenuated YopE-expressing bacteria (listed in figure legend (**Fig 7A and 7B, Fig 7A.1 and 7B.1**) or cholera toxin plus YopE_69-77_ peptide. Sixty days after immunization, mice were challenged with the mouse virulent *Y*. *pseudotuberculosis* strain YPIII/pIB1. Mice challenged via oral gavage, showed partial protection when immunized with any of the YopE expressing *L*. *monocytogenes* strains or the attenuated *Y*. *pseudotuberculosis* Δ*ksgA* strain (**Fig 7A and 7A.1**). Interestingly, mice challenged intravenously showed significant resistance to infection reflected in a prolonged mean day to death after being immunized with all YopE expressing *L*. *monocytogenes* strains or the attenuated *Y*. *pseudotuberculosis* Δ*ksgA* strain (**Fig 7B and 7B.1**). Also, a substantial increase in survival was observed in mice immunized with cholera toxin plus the YopE_69-77_ peptide (**Fig 7C and 7C.1**). These results suggest that a single immunization containing the immunodominant epitope of YopE can confer a partially protective CD8^+^ T cell driven host response against infection resulting in a delayed mean time to death.

## Discussion

In this study, we report that systemic immunization with a live-attenuated strain of *Y*. *pseudotuberculosis* generates YopE_-_specific CD8^+^ T cells. Immunization of mice with the *Y*. *pseudotuberculosis* live-attenuated *ksgA-* strain was sufficient to protect against challenge with virulent *L*. *monocytogenes* expressing YopE. Although not directly tested in this study, this protection was presumably dependent on YopE-specific CD8+ T-cells. Moreover, immunization with live-attenuated *L*. *monocytogenes* expressing YopE also results in the generation of YopE-specific CD8^+^ T cells and confers partial protection against lethal *Y*. *pseudotuberculosis* oral and systemic infection. In the case of immunization with *L*. *monocytogenes* expressing YopE, we show protection requires YopE-specific CD8+ T-cells. Our results indicate that *Yersinia* outer proteins can stimulate protective CD8^+^ T cell mediated immunity. Understanding the basis of immunity to *Y*. *pseudotuberculosis* is an important step in development of effective plague vaccines since *Y*. *pestis* is very closely related to *Y*. *pseudotuberculosis* [25].

The majority of work developing vaccines against *Yersinia* has focused on vaccines against *Y*. *pestis* to protect against plague. These vaccines have largely targeted the TTSS protein LcrV and the F1 protein [26]. Antibodies against these proteins provide significant protection against *Y*. *pestis* infection in a variety of animal models of plague [27–29]. A F1-LcrV fusion protein, F1V, is currently in late-stage development as a subunit vaccine against plague. In addition, to subunit vaccines targeting F1 and LcrV, a variety of approaches and vectors expressing these antigens provide protection against *Y*. *pestis* [26, 30–33]. Consistent with data presented in this study, we showed years ago that a live-attenuated *Y*. *pestis* vaccine provided significant mucosal and systemic protection against high-titer *Y*. *pestis* challenge [34]. Even though significant efforts and progress has been made in the preclinical development of the F1V vaccine, this vaccine has potential limitations. The F1 protein is not essential for virulence and natural and engineered strains of *Y*. *pestis* lacking F1 are virulent [35]. Therefore, identification of additional protective antigens or epitopes that can be included in future plague vaccine formulations is warrented. Although antibodies can provide protection against *Y*. *pestis*, it is now clear that T-cell mediated responses can contribute significantly to vaccine mediated protection. YopE is an attractive T-cell antigen since YopE is required for virulence and it generates very strong CD8^+^ T-cell responses.

*Yersinia* species are predominantly extracellular pathogens *in vivo* with many of their Yop-mediated virulence mechanisms targeting phagocytes by interfering with phagocytosis [8]. However, we and others provide evidence that immune responses generally associated with intracellular pathogens can be effective against *Yersinia* [9–12, 14, 29]. In this study, we took advantage of a recipricol heterologous vaccine/challenge system using *Listeria* and *Yersinia*. This allowed us to focus on the contributions of YopE-mediated immunity and in the case of immunizations with *Yersinia* expressing YopE and challenge with YopE-expressing *Listeria* we have a system where immunity is dependent on CD8+ T-cells. Altogether, data presented in this study suggests that CD8+ T-cells provide some protection against oral challenge with *Yersinia* and significantly delay the mean day to death in an intraveneous challenge model.

We recently demonstrated that mice immunized with live-attenuated *Listeria* expressing OVA were significantly protected from challenge with a virulent *Y*. *pseudotuberculosis* strain expressing a YopE-OVA fusion [11]. Protection required ova-specific CD8^+^ T-cells and perforin. These data were consistent with our earlier studies showing a general requirement for CD8^+^ T-cells and perforin to protect against *Yersinia pseudotuberculosis* [9]. This study suggested that CD8^+^ T-cells killed *Yersinia*-associated antigen presenting cells (APC) in a perforin-dependent manner. Bystander phagocytes can then engulf *Yersinia*-associated APCs without interference from the anti-phagocytic Yops. Others have presented evidence that perforin is despensible for CD8+ T-cell-mediated protection from *Y*. *pestis* but CD8 T-cell derived TNF-alpha and IFN-γ are critical [36]. It has become clear that cellular immunity to *Yersinia*, including CD8^+^ T-cell-mediated immunity, can provide significant protection against infection. Smiley and co-workers defined an immunodominant epitope in YopE, YopE_69-77_, that leads to robust generation of a functional CD8^+^ T-cell response capable of protecting mice against challenge with *Y*. *pestis* [37]. Consistent with the data presented in the current study, Bliska and co-workers demonstrated that YopE-specific CD8^+^ T-cells provide varying levels of protection against challenge with *Yersinia* [12]. We extend these studies by showing that immunization with the live-attenuated *ksgA* mutant induces the expansion of YopE-specific CD8^+^ T-cells that can provide significant protection against a systemic lethal heterologous challenge with *Listeria monocytogenes* expressing YopE. These data demonstrate that the protection is YopE-mediated as vaccinated control animals succumb to challenge with the parental *L*. *monocytogenes* strain and protection requires CD8+ T-cells. Consistent with findings observed with mucosal immunization with the immunodominant YopE epitope and Cholera toxin and subsequent *Y*. *pestis* challenge [10], mucosal immunization with YopE significantly protects against virulent mucosal challenge with *Y*. *pseudotuberculosis* (this work and [14]). Furthermore, vaccinated animals treated with an antibody that depletes CD8+ T-cells succumb to infection whereas animals treated with an isotype control antibody are protected strongly suggesting CD8+ T-cells are responsible for protection.

Although not directly tested in this study, we have previously shown that perforin expression by CD8^+^ T-cells is critical for controlling *Yersinia pseudotuberculosis* infection [9, 11] but others have not seen a requirement for perforin in protecting animals from *Y*. *pestis* [36]. Taken together, our work and that of others strongly suggest that the cytolytic functions of CD8^+^ T-cells in addition to their ability to produce IFN-γ and TNF-alpha are critical to control *Yersinia* infection. These observations could impact the design of future vaccines targeting *Y*. *pestis* and other *Yersinia* species.
